# Regional Dominant Frequency: A New Tool for Wave Break Identification During Atrial Fibrillation

**DOI:** 10.3389/fcvm.2018.00079

**Published:** 2018-06-25

**Authors:** Mohammad Hassan Shariat, Javad Hashemi, Saeed Gazor, Damian P. Redfearn

**Affiliations:** ^1^Department of Medicine, Queen's University, Kingston, ON, Canada; ^2^School of Computing, Queen's University, Kingston, ON, Canada; ^3^Department of Electrical and Computer Engineering, Queen's University, Kingston, ON, Canada

**Keywords:** atrial fibrillation, wave break, dominant frequency, signal processing, catheter ablation, electroanatomic mapping

## Abstract

Cardiac mapping systems are based on the time/frequency feature analyses of intracardiac electrograms recorded from individual bipolar/unipolar electrodes. Signals from each electrode are processed independently. Such approaches fail to investigate the interrelationship between simultaneously recorded channels of any given mapping catheter during atrial fibrillation (AF). We introduce a novel signal processing technique that reflects regional dominant frequency (RDF) components. We show that RDF can be used to identify and characterize variation and disorganization in wavefront propagation- wave breaks. The intracardiac electrograms from the left atrium of 15 patients were exported to MATLAB and custom software employed to estimate RDF and wave break rate (WBR). We observed a heterogeneous distribution of both RDF and WBR; the two measures were weakly correlated (0.3; *p* < 0.001). We identified locations of AF or atrial tachycardia (ATach) termination and later compared offline with RDF and WBR maps. We inspected our novel metrics for associations with AF termination sites. Areas associated with AF termination demonstrated high RDF and low WBR (↑RDF,↓WBR). These sites were present in 14 of 15 patients (mean 2.6 ± 1.2 sites per patient; range, 1–4 sites), 43% situated within the pulmonary veins. In nine patients where AF terminated to sinus rhythm (6) or ATach (3), *post-hoc* analysis demonstrated all ↑RDF,↓WBR sites were ablated and correlated with AF termination sites. The proposed RDF signal processing tools can be used to identify and quantify wave break, and the combined use of these two novel metrics can aid characterization of AF. Further prospective studies are warranted.

## 1. Introduction

Atrial fibrillation (AF) is the most common arrhythmia and a primary cause of stroke (1). It is characterized by heterogeneous spatiotemporal wavefront propagation that results in complex signal formation and fragmentation (2, 3).

Information extracted from intracardiac electrograms (IEGMs) collected from both atria, e.g., relative timing of pulmonary vein potentials, duration of bipolar electrograms (4), complex fractionated electrograms (CFE) (5), CFEmean (6), and the dominant frequency (DF) (7), are used to guide ablation therapy procedures; however, the underlying mechanisms for AF remain elusive and outcomes remain suboptimal in both paroxysmal (8) and persistent AF (9).

Thus, there is a need to better understand the sophisticated mechanisms around AF perpetuation. The relative delays between the activation times (ATs) of a mapping catheters electrodes are time variant in AF representing dynamic wavefronts as they pass a stationary catheters position (10). Described herein is a novel approach which encompasses the relationship between simultaneously recorded IEGMs and reflects the wavefront characteristics of a region through analysis of short term change in regional dominant frequency (RDF). Processing electrograms using a long time window can provide an accurate representation of stationary dominant frequency (11), whereas, shorter time windows, considered here, are more suitable for tracking transient changes in the dominant frequency. We developed a novel signal processing tool to quantify these changes and hypothesize mechanistic significance by correlation with clinical data derived from catheter ablation procedures for AF.

## 2. Methods

This study was carried out in accordance with the recommendations of the institutional ethics committee of Queen's University. The protocol was approved by the Queen's University Health Sciences and Affiliated Teaching Hospitals Research Ethics Board. Patients classified as paroxysmal or persistent who presented to the electrophysiology laboratory for a catheter ablation procedure with AF sustained for at least 7 days were eligible for inclusion. Antiarrhythmic drugs (other than Amiodarone) were held 5 half-lives prior to the study. The left atrium was mapped prior to ablation using an electroanatomic mapping (EAM) system (EnSite™ Velocity™ system, St Jude Medical, MN) and a circular mapping catheter with high electrode density, either a Reflexion™ HD or Reflexion™ Spiral (St. Jude Medical). Each catheter has 20 electrodes and a diameter of 25 mm; the bipolar pair electrodes spacing for Reflexion™ HD and Reflexion™ Spiral are 2 and 1 mm, respectively. The EAM color CFEmean map was employed to ensure ample sampling and even coverage of the endocardial surface and for the purposes of ensuring adequate contact and data acquisition. Data collection was carried on while patients were in AF and each region was sampled for 30 s. In two patients, recordings of 1 min were obtained in order to establish a minimum duration required for reproducibility (see sections 4.2 and 4.3). The data were recorded as segments with the sampling frequency of 2034.5 Hz and later exported to be used offline in the MATLAB (Mathworks, Natick, MA) environment for signal processing as described in section 3.

Catheter ablation was performed using radiofrequency energy (TactiCath™ Quartz) aiming for a force time integral (FTI) of 400 gs and maximal temperature of 43°C using 25–30 Watts power setting at each location. The ablation strategy was pulmonary vein isolation (PVI) using wide area circumferential lesions at least one centimeter from the pulmonary vein ostia with evidence of bidirectional block in sinus rhythm as an endpoint. A step wise approach was done employing Ibutilide 1mg infusion during geometry creation as an initial step, followed by additional substrate ablation based on a CFEmean map (12). Areas with CFE mean < 120 ms were tagged (6) and ablation delivered at areas representing the shortest cycle length first, with abolition of local electrogram and an FTI of 400 gs as the endpoint. Atrial tachycardias (ATach) arising during ablation were mapped using activation mapping and entrainment and ablated to restore sinus rhythm. No additional right atrial ablation was performed, and for patients classified as paroxysmal AF, no Ibutilide was administered.

Locations of AF or ATach termination were labeled on the EAM during the procedure; the location of AF termination was compared with MATLAB generated maps and correlated subjectively by overlay of maps using a research feature (Remote Maps, St. Jude Medical, MN). Patients were followed at 6 weeks, 3 months, 6 months and annually thereafter with 24 h Holter monitoring at each time point. Procedural outcomes were reported as both immediate (termination of AF) and recurrence of atrial arrhythmia during follow up defined as documented episode greater than 30 s or patient reported symptoms of over 30 min duration pending documentation. Patients reporting symptoms were provided with long term monitoring by event monitor or 7 day Holter monitors. Documented recurrences were classified as AF or ATach by surface electrocardiogram recording. During algorithm development, the engineers and physicians were blinded to procedural details or outcome.

## 3. Algorithm development

### 3.1. Regional dominant frequency and wave break rate

The dominant frequency from each bipole electrode pair of the spiral catheter was extracted from the frequency analysis of the preprocessed IEGM of that electrode pair independent of the rest of the catheters electrodes; to estimate the instantaneous electrode pair DF (iEDF), we first applied the preprocessing in (13) to the IEGM recorded from each electrode, removed the mean amplitude of the resulting signal, and estimated the iEDF from the extracted power spectrum. To obtain the power spectrum, short time Fourier transform (STFT) of the signal was calculated. For this aim, the signal was divided into segments with *T* = 1 s duration and 95% overlap; the Hanning window was applied on each segment and the power spectrum estimated using the fast Fourier transform (FFT). Finally, the iEDF of the *k*th electrode was calculated using ${\text{iEDF}}_{k}\left(t\right)=\arg\underset{f}{\max}P_{k}\left(f,t\right),$ where *P*_*k*_(*f, t*) is the power spectrum obtained from *k*th electrode of the catheter.

**Figure 1:**
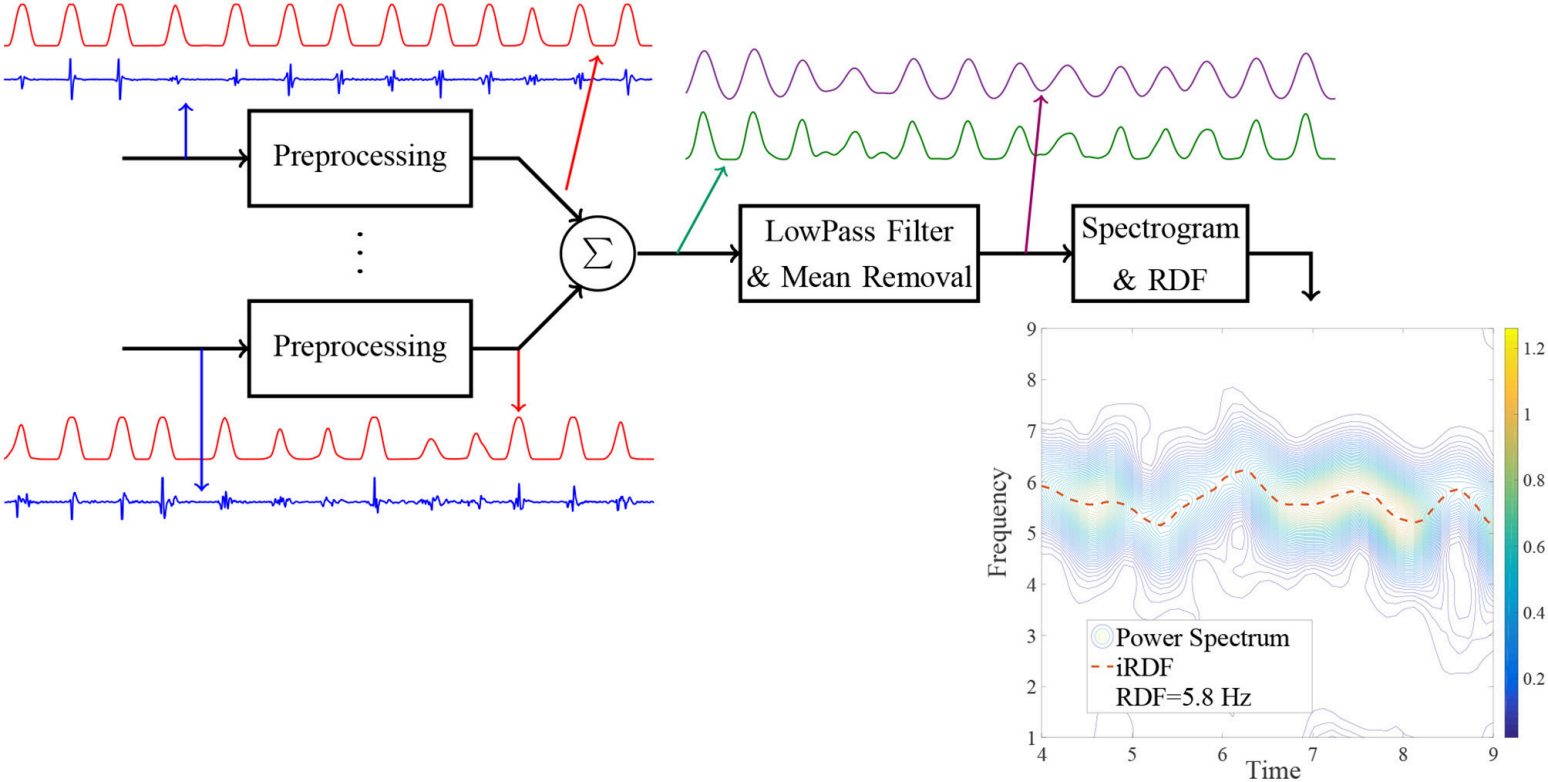
Block diagram of the proposed method for the regional dominant frequency (RDF) analysis and sample output of each stage.

To obtain the regional DF (RDF), first, the IEGM of each electrode pair of the catheter were preprocessed to generate a smooth train of pulses from the electrograms. Then, the preprocessed signals of all the catheter electrode pairs were averaged to produce one signal; this was smoothed by a lowpass filter, and the mean amplitude subtracted. Finally, the power spectrum of the resulting signal was used to estimate the DF, and the upper quartile extracted from the generated power spectrum reported as the regional DF. Figure 1, shows the flowchart of the proposed processing approach which includes sample output at each stage explained in more detail below. Similar to the iEDF calculation, in the first stage of the RDF calculation, the proposed method in (13) was applied to the IEGM recorded from each electrode pair of the catheter. This preprocessing step replaced the complex morphologies of the IEGMs with smooth Gaussian shape pulses. The AT of each bipolar electrode pair can be obtained by threshold crossing the associated preprocessed signal. However, here, we used the processed signal as an indicator of IEGM active intervals without trying to extract local ATs of electrodes which are prone to error (4). The preprocessed signals of all the electrodes were then averaged. The following two-sided exponential finite impulse response (FIR) filter (*h*) with the length of 180 is then used to further smooth the processed signal and allowed estimation of discontinuities in the wavefront propagation or wave break (WB):$h_{n}=\exp\left(-|-1+\frac{n-1}{90}|\right),\;\text{for}\;n=1,\cdots \;,180.$

The mean amplitude of the resulting signal was removed and in the next stage, the STFT of the signal calculated in a similar manner to the iEDF to obtain its power spectrum, i.e., the signal was divided into segments with *T* = 1 s duration and 95% overlap; the Hanning window was applied on each segment and the power spectrum of the signal estimated using the FFT. The instantaneous RDF (iRDF) and upper quartile of iRDF, (denoted as RDF), were calculated from the extracted power spectrum, i.e.

$$\begin{array}{rcl} \text{iRDF}\left(t\right) & = & \arg\underset{f}{\max}P\left(f,t\right),\\ \text{RDF}\left(D\right) & = & \underset{t\in \left[0,D\right]}{\text{upper quartile}}\text{ iRDF}\left(t\right),, \end{array}$$

where *D* is the duration of the IEGM segment, and *P*(*f, t*) is the power spectrum of the output of the 2-sided exponential lowpass filter.

We selected the time window length *T* in the STFT to be equal to 1 s; this value is smaller than the common value that is used for iEDF calculation (11, 14). The small value for *T* increased time resolution of the extracted iRDF and enabled us to identify wave break. Increasing *T*, however, increases the frequency resolution and degrades the time resolution thus obscuring transient wave break events (10, 15). We empirically defined wave break as any drop in the iRDF more than 3 Hz below the RDF (or below 0.5 Hz) and lasted longer than 100 ms, based upon expert consensus. Finally, the number of the wave breaks per second or the wave break rate (WBR) was proposed as a feature/measure to quantify the quality of the wavefront propagation at each site.

For the purposes of analysis, both RDF and WBR were assigned color on atrial geometries within the MATLAB environment. Sixty four colors were produced from the lowest recorded value to the highest value for each dataset producing a relative scale in each patient map. The highest and lowest recorded values were assigned red and blue colors, respectively.

#### 3.1.1. Example of RDF-based wave break identification

**Figure 2:**
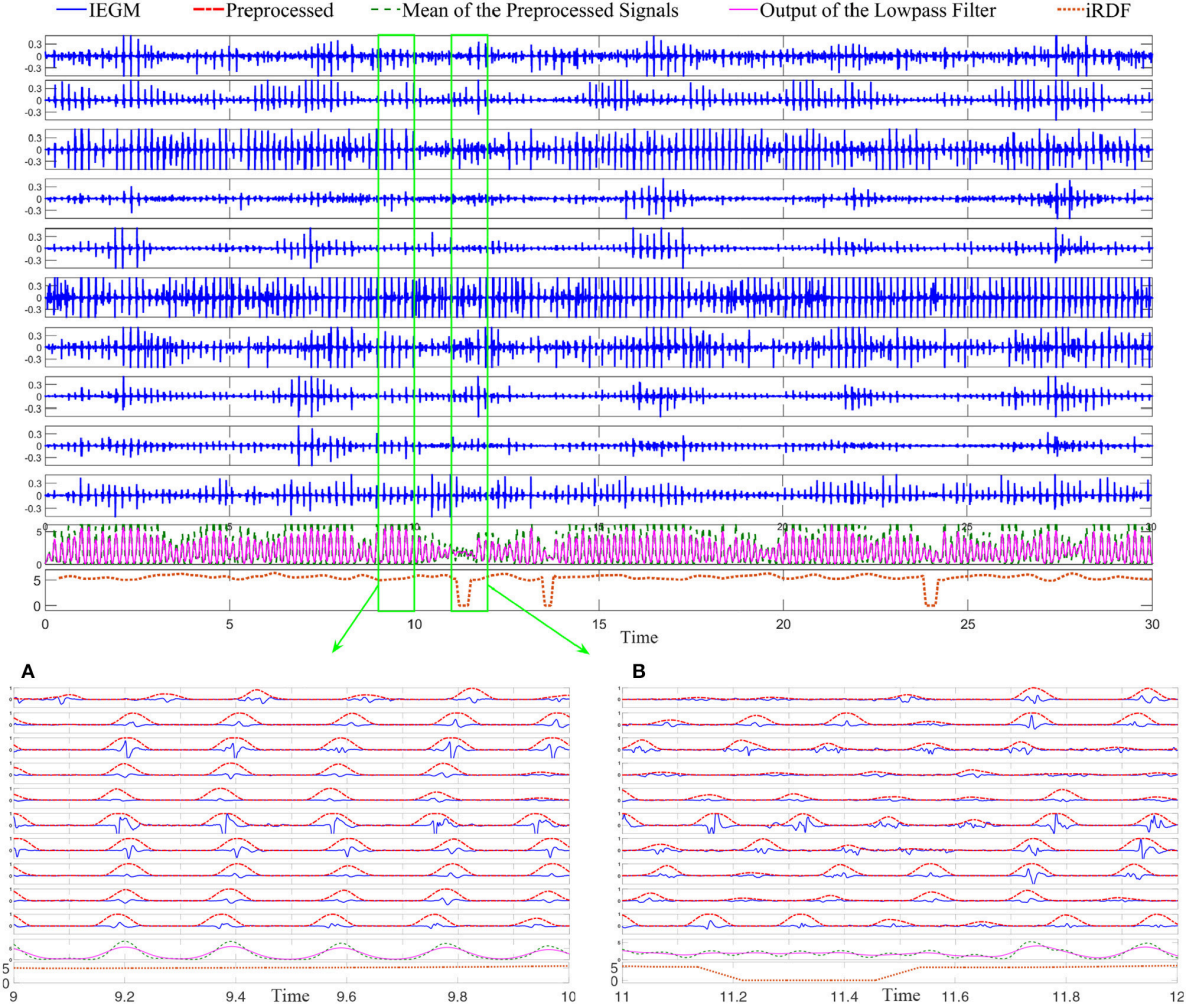
Bipolar intracardiac electrograms (IEGMs) collected using the Reflexion™ Spiral (St. Jude Medical) catheter from a patient with persistent AF. From top to bottom, axes 1 to 10, show the bipolar IEGMs. The average of the preprocessed signals and the low-passed version of that are plotted on the 11th axis; the 12th axis shows the dominant frequency (DF) of the lowpassed signal. Subfigure **(A)** shows the IEGM segment with clear wavefronts; the normalized preprocessed signals (with the maximum amplitude of one) are also plotted in this sub-figure. The output of the lowpassed filter has a large peak for each wavefront and the iRDF for this subfigure varies between 4.9 and 5.5 Hz. Subfigure **(B)** shows another segment of the IEGM with a wave break in which there are no distinguishable wavefronts at the beginning of the segment. Here, the average of the preprocessed signal has multiple small peaks that are not present in the lowpassed version of it, and there is a significant drop in the iRDF at this time.

Figure 2 shows sample EGMs recorded from the roof of the left atrium of a patient with persistent AF using the Reflexion Spiral catheter; the outputs of various stages of the proposed processing are plotted for this segment. For the regions where a clear wavefront was present, the peaks of the pre-processed signals of all the electrodes occurred very close to each other; therefore, the averaged signal generated a large peak for each wavefront; see Figure 2A. However, for the time intervals when a wave break occurred, the delays between the ATs of the electrodes increased and, consequently, the peaks of the pre-processed signals occurred over a longer time interval. In this case, averaging the pre-processed signals generated several small peaks resulting in a segment with high frequency. This high frequency component of the signal was attenuated significantly by the lowpass filter leading to a drop in the iRDF; see Figure 2B. This example shows how changes in iRDF can be used to study the wavefront variation and identify wave breaks. Three wave breaks were present in this EGM segment (at around Time = 12 and 24 s), and the WBR for this segment is estimated as 0.1 WB/s.

**Figure 3:**
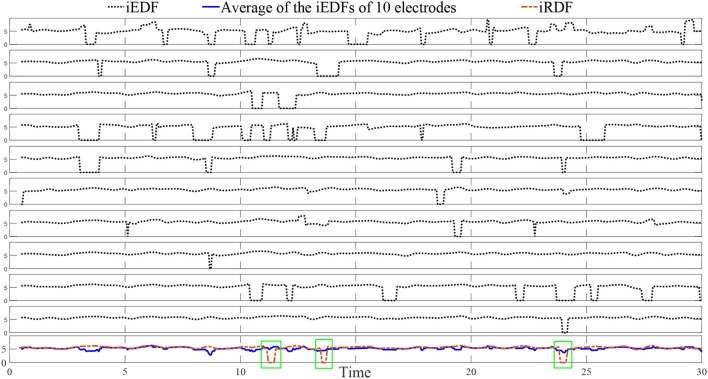
The instantaneous electrode dominant frequency (iEDF) and the instantaneous regional dominant frequency (iRDF) of the IEGM shown in Figure 2 when *T* = 1 s. The average of the iEDF of all the electrodes (shown in blue) does not drop at the same time as the iRDF, identifying different wave break (WB) instances. Green boxes mark WB instances obtained using iRDF.

Figure 3 demonstrates 10 iEDFs of the electrograms shown in Figure 2; the average of all the iEDFs of the catheter is shown below (blue line) compared with iRDF. Note that the iRDF in Figure 2 is different from the average of the iEDFs of the electrodes of the catheter exposing WB not shown by the average iEDF.

### 3.2. Statistics

The Anderson-Darling test was used to inspect for normality. Non-parametric data was compared using the Mann-Whitney test and was used to compare the WBR and RDF of persistent and paroxysmal patients. Spearmans rank correlation coefficient used to study the correlation between WBR and RDF (*p*-value less than 0.05 is considered statistically significant). The mean and standard deviation of variables are reported using the mean ± std notation.

## 4. Results

### 4.1. Demographics

Twenty consecutive patients were recruited, five patients were excluded due to poor data quality and incomplete coverage of the left atrial chamber defined as endocardial surface coverage of less than 60% (the uncovered area included the mitral valve and pulmonary vein ostia.). Average procedural duration for the remaining 15 patients was 4:39 ± 0:54 h (persistent 4:53 ± 0:42 h, paroxysmal 4:12 ± 1:09 h). There were 5 paroxysmal and 10 persistent patients (mean duration of persistent AF 20.6 ± 8.6 months), 13 males, mean age was 61.3 ± 9.2 years. The mean left atrial diameter was 47 ± 9.4 mm (persistent 50.9 ± 7.8 mm and paroxysmal 39.2 ± 7.7 mm). There was an average of 24.4 ± 7 recording locations (488 ± 140 unipolar electrograms/mapping points) per patient and mean recording duration of 29.9 ± 9.8 s. In 8/10 persistent patients Ibutilide 1mg was administered prior to sampling of the AF electrograms. Two patients were taking Amiodarone.

### 4.2. Minimum required segment duration for accurate RDF estimation

**Figure 4:**
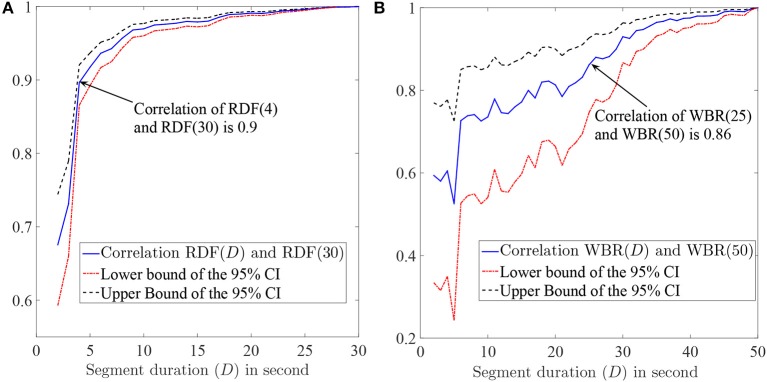
**(A)** The Pearson correlation of the RDF(*D*) and RDF(30) and also the upper and lower bounds of the 95% confidence interval (CI) of the correlation as a function of segment duration *D*. **(B)** The Pearson correlation between the wave break rate (WBR) obtained using the *D*-second segment and the one obtained from the 50 s segment. The 95% CI bounds of the correlation are also plotted. The value in parenthesis in front of WBR or RDF shows the electrogram duration (in second) that is used to calculate corresponding feature.

We aimed to find the minimum segment duration that was required for an accurate and robust estimation of the RDF. We assumed that the data obtained using a 30-s segment was the gold standard, our goal was to find the segment duration such that the Pearson correlation between the desired feature obtained from that segment and the gold standard was higher than 85%. We selected EGMs of patients with durations longer than 30 s (201 segments/sites were selected from 15 patients) and calculated the RDF(30) for them. We also calculated the RDF using shorter segment durations *D* (only first *D* seconds of IEGMs were used) and compared the results. Figure 4A shows the Pearson correlation between the RDF(*D*) and the RDF(30) for various *D*s; the upper and lower bounds of the 95% confidence interval (CI) of the correlation are also plotted. From this figure, we concluded that the RDF obtained using the EGM segment longer than 4 s is an accurate estimate of the RDF(30), as the correlation of the RDF(4) and RDF(30) is 90%.

### 4.3. Minimum required segment duration for accurate WBR estimation

Having established the use of 4 s segments provided an accurate estimation of the RDF, we established the minimum required segment duration for reliable WBR estimation. We followed the same procedure as described in the previous section. Segments with duration longer than 50s (37 segments) were selected from two patients, and for each segment, the WBR was obtained using the first 50 s of each segment. The Pearson correlation (and the 95% CI bounds) of the WBRs estimated from *D*-second segments and 50 s segments are plotted in Figure 4B. Based on this figure, we concluded that IEGM segments longer than 25 s are required for reliable estimation of the WBRs.

### 4.4. Regional dominant frequency and wave break rate

Stable segments longer than 25 s were selected from 15 patients (279 segments). The first 25 s of each segment was used for the RDF and, consequently, the WBR estimation. The mean RDF of the segments was 5.5 ± 0.82 Hz (median 5.4 Hz; range, 2.86–7.66 Hz), and the WBR was 0.16 ± 0.13 WB/s (median 0.15 WB/s; rang, 0 to 0.63 WB/s). The RDF and WBR for the five paroxysmal patients was 5.99 ± 0.8 Hz (median 5.94; range, 3.47–7.66 Hz) and 0.24 ± 0.14 (median 0.23; range, 0–0.63 WB/s) respectively. For the 10 persistent patients, the RDF and WBR was 5.32 ± 0.75 Hz (median 5.27; range, 2.86–7.03 Hz) and 0.14 ± 0.11 (median 0.13; range, 0–0.47) respectively. The difference was significant (*p* < 0.001) for both RDF and WBR.

**Figure 5:**
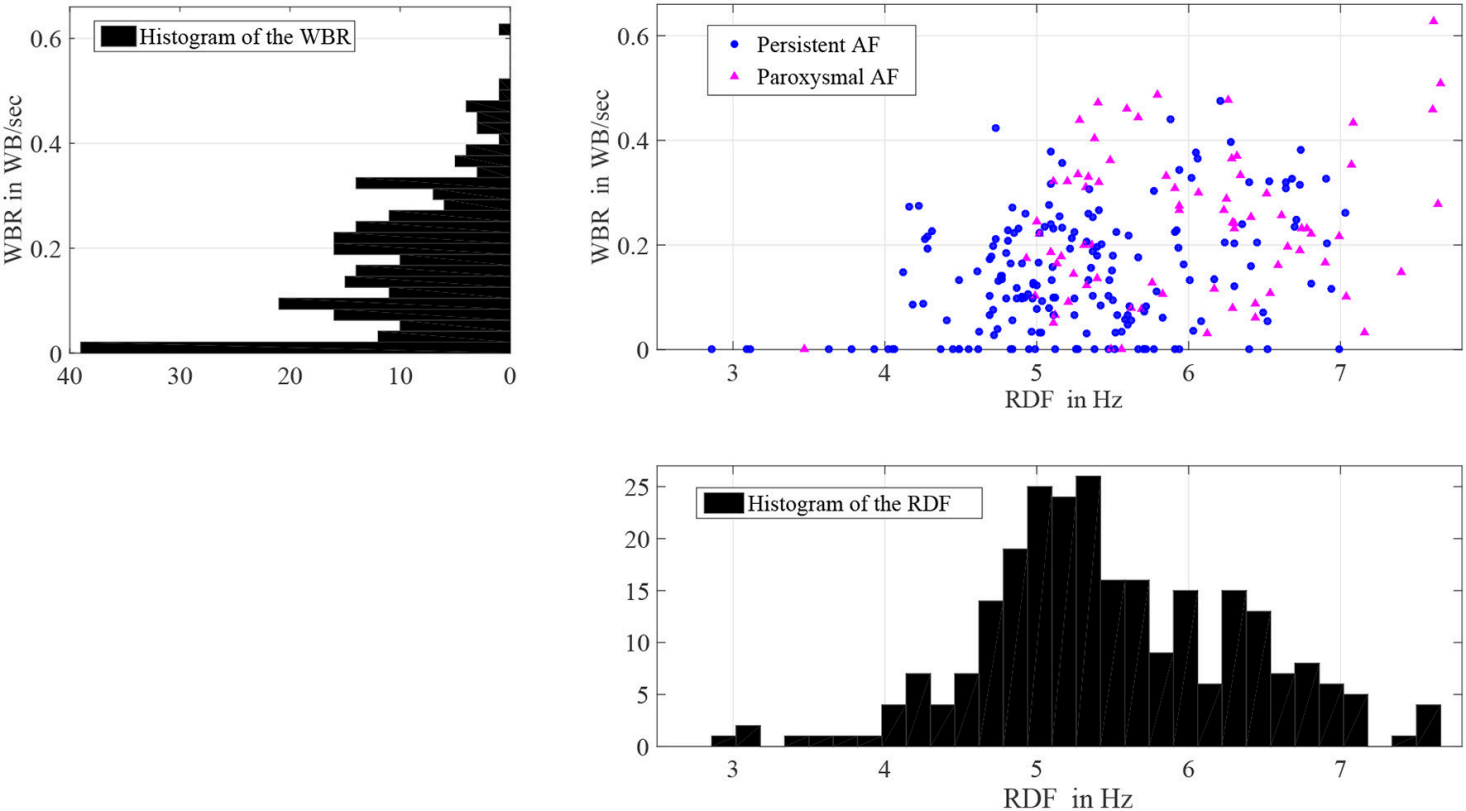
Histograms of the wave break rate (WBR) in WB/s and the regional dominant frequency (RDF) in Hz, and also the scatter plot of the WBR and RDF for 258 segments with durations longer than 25 s.

**Figure 6:**
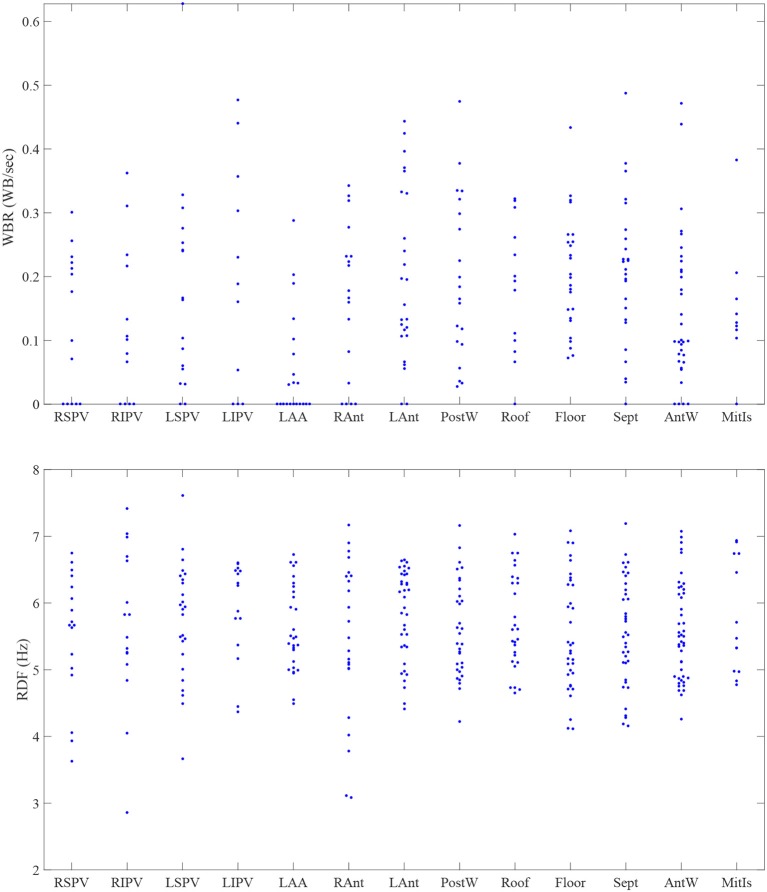
The estimated values for the wave break rate (WBR) in WB/s and the regional dominant frequency (RDF) in Hz in different left atrial sites. RSPV, right superior pulmonary vein; RIPV, right inferior pulmonary vein; LSPV, left superior pulmonary vein; LIPV, left inferior pulmonary vein; LAA, left atrial appendage; RAnt, right antrum; LAnt, left antrum; PostW, posterior wall; Sept, septum; AntW, anterior wall; MitIs, Mitral Isthmus.

There was significant heterogeneity in distribution of WB and RDF, the two measures were weakly correlated (0.3; *p* < 0.001). Figure 5 shows the scatter plot of the estimated WBR and RDF, in which circles and triangles are used to mark the estimated values from the patients with persistent and paroxysmal AF, respectively. The histograms of the WBR (in WB/s) and RDF (in Hz) are also shown. Figure 6 shows the estimated values for the WBR and RDF at different left atrial sites for all the segments collected from the patient cohort. There was a trend toward relatively low WBR in the left atrial appendage and both the anterior wall and veins, but overall there was non-significant variation in relative values across the LA geometries.

### 4.5. Procedural outcomes and RDF/WBR correlation

**Figure 7:**
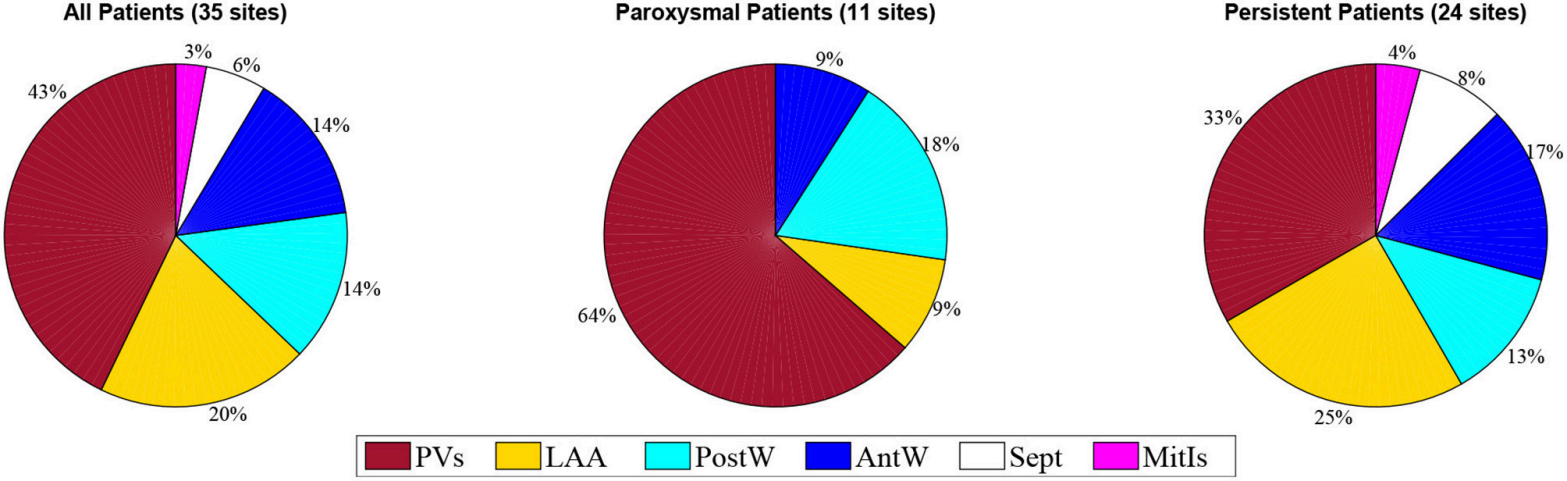
The prevalence of the ↑RDF-↓WBR sites at different anatomical positions in the paroxysmal and persistent patients. PVs, pulmonary veins; LAA, left atrial appendage; PostW, posterior wall; Sept, septum; AntW, anterior wall; MitIs, Mitral Isthmus.

Of 15 patients, ablation terminated AF to sinus rhythm in six patients and ATach in three; a further six patients underwent direct current cardioversion (CV) at the discretion of the operator. We marked sites where termination of AF occurred with ablation on the geometries populated with RDF and WBR data. Sites of high RDF and low WBR (↑RDF,↓WBR) were defined as sites where RDF and WBR were in the upper and lower quartile range of the calculated values for each patient, respectively. Thus, a ↑RDF,↓WBR site was described based on the relative values in each patient and not absolute values; these sites were identified in 14/15 patients (2.6 ± 1.2 sites per patients; range, 1–4 sites; 43% situated within the pulmonary veins). More specifically, 2.2 ± 1.48 sites with ↑RDF,↓WBR were observed in paroxysmal patient, where most of them (63%) were located in the pulmonary veins. For persistent patients, 2.4 ± 1.17 sites with ↑RDF,↓WBR were observed that only 33% were located at PVs (see Figure 7 and Table 1).

**Table 1:** Patient demographic, procedural data, and outcome.

**Gender**	**Age**	**Persistent AFduration (months)**	**LA diameter(mm)**	**Number of↑RDF-↓WBR**	**All ↑RDF-↓WBRAblated**	**Outcome of procedure**	**↑RDF-↓WBR siteof termination**	**Recurrence**
M	62	0	34	4	1	CV to SR	NA	0
M	77	24	57	3	0	CV to SR	NA	AF
M	60	10	55	4	0	CV to SR	NA	0
M	80	18	47	3	0	CV to SR	NA	AF
M	67	28	54	1	1	CV to SR	NA	ATach
M	49	18	52	4	0	CV to SR	NA	ATach
M	60	0	31	3	1	ATach	1	0
M	54	24	52	2	1	ATach	1	0
M	56	36	65	2	1	ATach	1	ATach
M	49	6	38	1	1	SR	1	0
F	59	0	39	0	NA	SR	NA	0
M	65	0	51	2	1	SR	0	0
M	60	18	41	3	1	SR	0	0
F	69	24	48	1	1	SR	1	0
M	52	0	41	2	1	SR	1	0

*Value 0 in “persistent AF duration” column represents paroxysmal patient. AF, atrial fibrillation; LA, left atrium; M, male; F, female; CV, cardioversion; SR, sinus rhythm; ATach, atrial tachycardia; NA, not applicable*.

Nine patients had termination of AF with ablation, six to sinus rhythm and three to Atach; ↑RDF,↓WBR sites were present in eight of these nine. In five of the six patients where AF terminated to sinus rhythm, ↑RDF,↓WBR sites were present and ablated. In all three patients where AF terminated to ATach we observed ↑RDF,↓WBR sites during AF at the site of subsequent ATach termination. In one patient no ↑RDF,↓WBR site was observed; this patient has a history of paroxysmal AF and terminated to sinus rhythm during right pulmonary vein antral ablation.

Mean follow-up was 24.5 ± 6.3 months (range 17–32 months). When we examined the six patients that underwent cardioversion, only two patients had all sites with ↑RDF,↓WBR ablated; during follow up of those patients, four had recurrence, ATach (2) and AF (2). No recurrence was reported in the cohort of six patients where ablation successfully terminated AF to sinus rhythm; whereas, among three patients that terminated to ATach, one patient had ATach recurrence. Number of ↑RDF,↓WBR sites were larger among patients requiring CV to sinus rhythm (SR) compared to the rest of the patients (3.17 ± 1.17 vs. 1.78 ± 0.97 sites).

**Figure 8:**
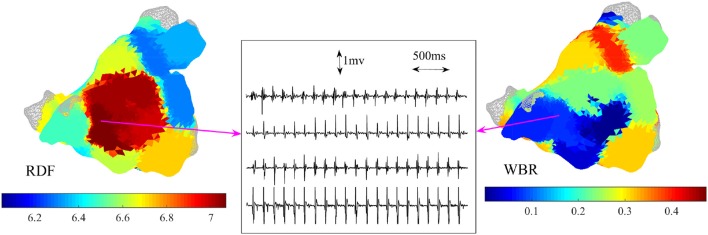
Example of 3-dimensional atrial maps color coded based on the regional dominant frequency (RDF) and wave break rate (WBR). Four bipolar IEGMs of the catheter at a site with high RDF (7 Hz) and low WBR are shown.

**Figure 9:**
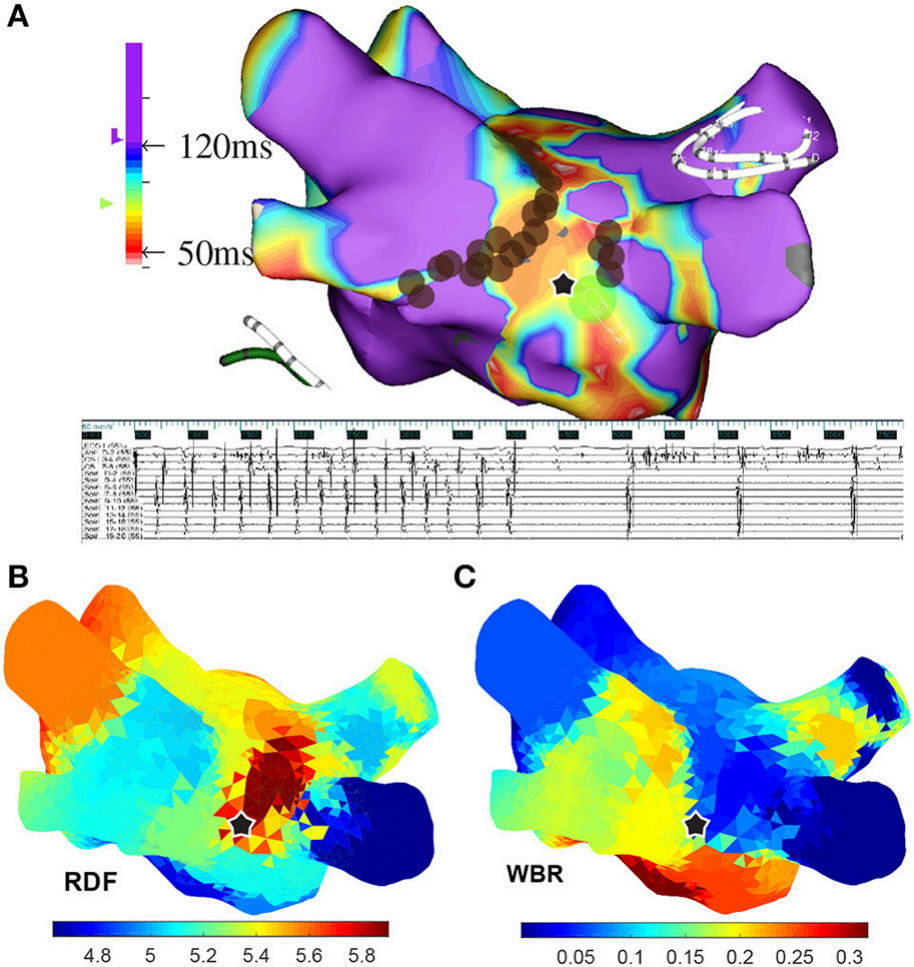
**(A)** A 3-dimensional atrial map color coded based on the CFEmean for a patient with persistent AF; the ablation sites are marked with brown circles. **(B)** From top to bottom surface ECG (lead I), IEGMs of the ablation catheter placed at the location marked with star, collected from the Inquiry™ (St. Jude Medical) catheter placed in coronary sinus (CS), and from Reflexion™ Spiral (St. Jude Medical) placed in right superior pulmonary vein. **(C)** Atrial maps color coded based on the regional dominant frequency (RDF) and wave break rate (WBR) when 24 segments longer than 25 s are used to create these maps. In all the atrial maps, the ablation site that lead to AF termination is marked with black star.

Table 1 shows these results and Figures 8, 9 are illustrative of the concept. Figure 8 shows a sample ↑RDF,↓WBR site. Four electrograms from this site are also shown in this figure. Figure 9 highlights cumulative ablation lesions that associated with termination of AF to SR on a geometry color coded for CFEmean in a patient with persistent AF; the area just posterior to the right inferior pulmonary vein (RIPV) can be observed to present high RDF and low WBR with an area immediately inferior showing a high WBR. As the ablation line crosses this region the AF terminated.

## 5. Discussion

Traditional electrogram and anatomical-guided ablation (pulmonary vein isolation, empiric lines, CFEmean, EDF) is not a satisfactory treatment for persistent AF (9, 16). Many operators have observed AF termination at fractionated sites, and targeting fractionation was conceptually supported by some authors (17, 18). However, mapping of fractionated signals using CFEmean values has not delivered the success expected due to the numerous confounding factors (19, 20). Wave break and collision are common findings in models of AF resulting in high frequency and complex electrograms (21) yet ablation of CFEmean appears no better than empiric ablation (9, 20). On the other hand, experimental work has suggested high frequency electrograms are located at sources of AF; these sources are surrounded by continuous wave break, potentially obscuring the driver (22). We hypothesized that wavefront dynamics might be objectively quantified and provide some insights into the mechanisms of complexity and AF drivers. To provide mechanistic insight we used termination of AF with ablation as an endpoint.

Our goal was to develop a computationally efficient algorithm to identify and quantify regional wavefront discontinuities, or wave breaks, for further characterization of AF patterns. We observed that when ablation approximated sites with high RDF and low WBR (↑RDF,↓WBR) there was an association with termination of AF; this is in keeping with experimental data on sources (23, 24).

Regional dominant frequency identified regions with rapid change in wavefront propagation. This was done without directly identifying the activation time at individual bipoles, as accurate local activation time estimation is very challenging (4). A recent paper from Zaman et al. elegantly exposed the problems with phase mapping and the reliability on activation times (25). The proposed method here was designed to be more robust and efficient for characterization of wavefronts with long/fractionated/non-discrete activations and without clear isoelectric lines between activations. Unorganized activations are the result of rotating waves, local conduction block, and wavefront collision (2, 26–29); quantifying the disorganization at wave breaks during the recordings from each atrial site informs AF mechanism and perpetuation (26).

While some studies purport to visualize “rotors” using a large basket catheter (30–32), there are numerous limitations with both the technology and translation of findings. All of these continuous mapping technologies (e.g., Rhythmia Mapping, Boston Scientific and CARTOFINDER, Biosense Webster) require simultaneous acquisition of endocardial signals. They work best with less advanced substrate; and due to the large bipole spacing necessitated by basket construction, they lack resolution for low amplitude fractionated electrograms. Furthermore, lack of contact between the electrodes and the tissue is a considerable problem when a basket catheter is deployed (33). Our approach was to investigate the feasibility of sequential mapping using catheters with higher density of electrodes to inspect regions of the atria. This lacks the ability to map wavefronts across the atria in temporal relation with more distant regions and is time consuming; however, it does allow us to look for the “signature” of an AF source or rotor. For any focal ablation to be successful, it requires a relatively stationary or reproducible source; therefore, regional, sequential analysis might provide sufficient differences to discern a source from a passive wavefront or collision. We tested this hypothesis with careful analysis of sequential signals that were collected prior to ablation and later correlation with ablation lesion sets and outcome. A sequential approach would allow the operator more flexibility in data collection ensuring good contact throughout various atria and can use catheters already designed and capable of excellent geometry creation and pulmonary vein isolation.

The study of the spatial distribution of WBR and RDF might provide clinically important insight regarding putative sources of AF as suggested in these early data. The WBR is proposed as a feature/measure to quantify the qualities of the wavefront propagation at each site; it can be color-coded and shown on electroanatomic mapping systems and employed to characterize and differentiate signal complexity leading to the potential for a more informed choice of ablation target than current empiric techniques. Importantly the data is collected sequentially but used regionally, this provides improved endocardial resolution over panoramic surface ECG and current balloon based technologies. Our work has shown that 4 s is sufficient for RDF reproducibility at any site and the addition of WBR can be performed within 25 s of data collection. These early data suggest WBR and high RDF sites might differentiate putative sources from passive collision based on our correlation with ablation termination sites. With successful development and validation of these tools, further work is needed to characterize wave break and establish its relationship to AF sources and ablation outcomes.

## 6. Limitations

Averaging the pre-processed signals of the spiral catheter to create wavefront analysis is done at the expense of spatial resolution, providing a feature value for a region of approximately 2.5 cm in diameter. Shorter recording times (4 s) will facilitate efficient identification of sites requiring longer recordings and overlapping will improve resolution.

We used termination of AF as a surrogate marker for identification of a critical area, or source, for AF perpetuation. Acute termination is an acceptable surrogate in many studies e.g., (31); however, we also used recurrence during follow-up to support these assumptions.

Ibutilide was used in 8/10 persistent patients and none of the 5 paroxysmal patients. The use of Ibutilide to facilitate catheter ablation is still debated; however, the potent class III effect will reduce mean AF cycle length and consequently RDF values (34). This may explain why the mean RDF for paroxysmal patients was significantly higher than for persistent, contrary to the available literature (7). Furthermore, electrograms were collected exclusively from the left atrium and the right atrium during AF is not represented in our analysis.

## 7. Conclusion

Traditional electrogram and anatomical-guided ablation (pulmonary vein isolation, lines, CFEmean, DF) is not a satisfactory treatment for persistent AF. Discontinuity of wavefront propagation during continuous AF might be the result of rotating waves, local conduction block, or wavefront collision. We introduced the novel RDF concept and showed that it can be used to identify and quantify unorganized activations that represent discontinuities in wavefront activation and, thus, provide insightful information that potentially can be used for catheter ablation therapy during human atrial fibrillation. Our method offers a novel metric for further investigation and understanding of atrial fibrillation.

## Author contributions

Study design, data acquisition, analysis and interpretation of data, and drafting the paper were done by MHS and DR. All authors contributed in editing and approving the final manuscript.

### Conflict of interest statement

The authors declare that the research was conducted in the absence of any commercial or financial relationships that could be construed as a potential conflict of interest. The reviewer SM and handling Editor declared their shared affiliation.
